# Predicting aptamer sequences that interact with target proteins using an aptamer-protein interaction classifier and a Monte Carlo tree search approach

**DOI:** 10.1371/journal.pone.0253760

**Published:** 2021-06-25

**Authors:** Gwangho Lee, Gun Hyuk Jang, Ho Young Kang, Giltae Song

**Affiliations:** 1 School of Computer Science and Engineering, Pusan National University, Busan, Republic of Korea; 2 Research & Development, NuclixBio, Seoul, Republic of Korea; Kingston University, UNITED KINGDOM

## Abstract

Oligonucleotide-based aptamers, which have a three-dimensional structure with a single-stranded fragment, feature various characteristics with respect to size, toxicity, and permeability. Accordingly, aptamers are advantageous in terms of diagnosis and treatment and are materials that can be produced through relatively simple experiments. Systematic evolution of ligands by exponential enrichment (SELEX) is one of the most widely used experimental methods for generating aptamers; however, it is highly expensive and time-consuming. To reduce the related costs, recent studies have used *in silico* approaches, such as aptamer-protein interaction (API) classifiers that use sequence patterns to determine the binding affinity between RNA aptamers and proteins. Some of these methods generate candidate RNA aptamer sequences that bind to a target protein, but they are limited to producing candidates of a specific size. In this study, we present a machine learning approach for selecting candidate sequences of various sizes that have a high binding affinity for a specific sequence of a target protein. We applied the Monte Carlo tree search (MCTS) algorithm for generating the candidate sequences using a score function based on an API classifier. The tree structure that we designed with MCTS enables nucleotide sequence sampling, and the obtained sequences are potential aptamer candidates. We performed a quality assessment using the scores of docking simulations. Our validation datasets revealed that our model showed similar or better docking scores in ZDOCK docking simulations than the known aptamers. We expect that our method, which is size-independent and easy to use, can provide insights into searching for an appropriate aptamer sequence for a target protein during the simulation step of SELEX.

## Introduction

Aptamers are tertiary structures composed of relatively short, single-stranded oligonucleotides or peptide fragments [1–3]. Aptamers possess various properties, such as small size, tissue/cell-penetrating capacity, low toxicity, low immunogenicity, and simplicity with respect to chemical modification [4]. Owing to the cost of components and complexity of the experimental steps, oligonucleotide-based aptamers are mainly used rather than peptide-based aptamers, and selective diagnosis or treatment of tumors is possible because of target specificity [5–7].

For many therapeutic applications, aptamers are typically generated from random combination libraries (approximately 10^16^ random RNA or DNA sequences) using systematic evolution of ligands by exponential enrichment (SELEX) [1, 2]. The SELEX process requires multiple rounds of incubation, binding, washing, target-bound elution, and amplification. Thus, it takes a few days to several months to generate an aptamer library [8].

To decrease the time and expense involved in *in vitro* aptamer selection, SELEX is combined with high-throughput sequencers, called HT-SELEX, and multiple computational methods have been developed for aptamer selection using HT-SELEX data [9]. AptaCluster [10] and FASTAptamer [11] are examples of such computational methods that are based on clustering of massive sequence pools derived from HT-SELEX. Chushak and Stone [12] introduce the following three steps for selecting a primary sequence pool for *in vitro* selection experiments: choosing RNA aptamers based on their secondary structure, three-dimensional structure modeling, and computational docking. Ahirwar *et al*. [13] implement an *in silico* method for aptamer selection using steps similar to those used by Chushak and Stone [12], including three-dimensional structure modeling and molecular docking simulation, which are related to analyzing sequences and aptamer-protein interactions (APIs).

Recently, several machine learning methods have been proposed to assess API pairs. Most studies have employed the API classifier, which performs a binary classification for determining the interaction or non-interaction of a given aptamer-protein sequence pair using sequences and additional sequence-derived features, such as pseudo K-tuple nucleotide composition [14, 15], discrete cosine transformation [16], disorder information, and bi-gram position-specific scoring matrix derived from PSI-BLAST [17]. Previous studies conducted by Li *et al*. [18] and Zhang *et al*. [19] have used the aforementioned features. Recently, RPITER [20] has designed a deep learning approach for the API classification using the primary and secondary structures of input sequences. Since these methods have focused on the API classification only, they are unable to generate aptamer sequences. Lee and Han [21] select potential aptamer candidates among randomly sampled sequences using a heuristic approach with an API classifier, but their method requires strict constraints (*e.g*., the length of the aptamer sequence was fixed at 27 bases). To resolve these issues, we propose a novel generative model as follows.

Building the generative model is similar to a machine translation problem. For an aptamer-protein sequence pair, a target protein can be regarded as a pre-translation sentence and an aptamer as a post-translation sentence within the machine translation. While Google’s neural machine translation uses a benchmark dataset that contains approximately 2 million sentences for training the translation model between English and French [22, 23], we have datasets that include only a few thousand API pairs. To the best of our knowledge, there is no end-to-end generative model for generating aptamer sequences that interact with a given target protein, in part because there is no sufficient data to train the generative model. To build the end-to-end generative model with limited training data, we have designed our generative model architecture using a discriminative model and a sequence sampler. The discriminative model is an API classifier that uses the sequences (RNA aptamer and protein) as input features. Multiple API classification models are trained based on the benchmark datasets that have been used in previous studies [18, 19, 21]. These trained API classifiers are applied to the sequence sampler. A search space for sampling random nucleotide sequences with fixed length *N* is 4^*N*^. It the sequence length is not fixed, the search space becomes even bigger. To permit aptamer sequences of variable lengths, we design the sequence sampler using an iterative sequence sampling algorithm based on the Monte Carlo tree search (MCTS) [24] and the API classification models (note that the API classification models are used as a score function for the MCTS algorithm). This enables our approach to mimic an end-to-end generative model using the fast and efficient search.

We call this iterative generative model with MCTS as Apta-MCTS. We generate candidate aptamer sequences for target proteins using Apta-MCTS, combine the aptamer candidates with target protein structures via a docking simulation tool, and evaluate our Apta-MCTS using docking scores from the simulation. Our validation shows that Apta-MCTS can generate potential candidate aptamer sequences *in silico* efficiently.

## Materials and methods

### Data preparation

There are several data sources that are commonly used for building an API classification model [18, 19, 21], including aptamer base [25] and Protein Data Bank (PDB) [26]. We used these data to train our prediction model and evaluate it. They contain both DNA- and RNA-binding aptamers that interact with target proteins. These are separated into training and test datasets. Both Lee and Han [21] and Li *et al*. [18] used the collection of experimental results for aptamer-protein complexes. While both methods determined interaction and non-interaction pairs, Lee and Han [21] examined the interaction in narrow resolution in terms of aptamer nucleotides and Li *et al*. [18] evaluated the same in broad resolution. Since these two datasets have different resolutions, we trained two separate models for identifying the API in both narrow and broad resolutions.

Table 1 presents a summary of these datasets. The dataset from Li *et al*. [18] was split into the training dataset, which contained 580 positive and 1740 negative aptamer-protein sequence pairs, and the test dataset, which contained 145 positive and 435 negative pairs of sequence nucleotides and proteins. Since T in DNA is similar to U in RNA, we treated the letter T (thymine) as U (uracil) before encoding as described previously [18]. This benchmark dataset was used for training and validating our API classifier. The dataset from Lee and Han [21] was also separated as the training dataset, containing 157 positive and 493 negatives, and the test dataset, containing 56 positive and 56 negative RNA pairs. While this benchmark dataset was used for API classification modeling as in the case of the benchmark dataset of [18], the positive 56 pairs in the test dataset of [21] were used for evaluating candidate aptamers of our model Apta-MCTS. Notably, 56 pairs in the test dataset were collected from the test dataset of Li *et al*. [18] with only RNA aptamers [21].

**Table 1:** The two benchmark API datasets that are used for building two different classification models.

Source	Number of positive pairs	Number of negative pairs	Description
[18]	580	1740	Training data for API classifiers
	145	435	Validation data for API classifiers
[21]	157	493	Training data for API classifiers
	56	56	Validation data for API classifiers and Apta-MCTS

Note that we obtained two pre-trained API classifiers for the aptamer generative model, Apta-MCTS, using the benchmarks.

### Data representation

To feed aptamer and protein sequences into our classification model, the sequences were encoded into a numerical representation. While most machine learning models generally use a feature vector of a fixed size as an input, aptamer and protein sequences are of variable lengths. To this end, various encoding methods have been applied to the protein and aptamer(DNA/RNA) sequences. To choose an optimal encoding function for protein and aptamer sequences, we applied all 54 combinations of encoding methods for our API classification models: 9 methods for aptamer sequences [Dinucleotide Auto-Covariance (DAC), Dinucleotide Cross-Covariance (DCC), DACC(DAC+DCC), Trinucleotide Auto-Covariance (TAC), Trinucleotide Cross-Covariance (TCC), TACC(TAC+TCC) [27], Psuedo K-tuple Nucleotide Composition (PseKNC) [15] where K = 2 and 3, and Improved Conjoint Triad Feature (iCTF) [20]], and 6 methods for protein sequences [Amino Acid Composition (AAC), DiPeptide Composition (DPC), TriPeptide Composition (TPC), Pseudo Amino Acid Composition (PseAAC) [14], Composition-Transition-Distribution (CTD) [28], and iCTF]. All encoding methods were implemented using the propy [29] and PyBioMed [30] packages except the iCTF which was downloaded from the RPITER GitHub repository. We compared the performance of all these encoding methods and chose an optimal encoding method.

### Training a model for API classification using a random forest approach

**Fig 1:**
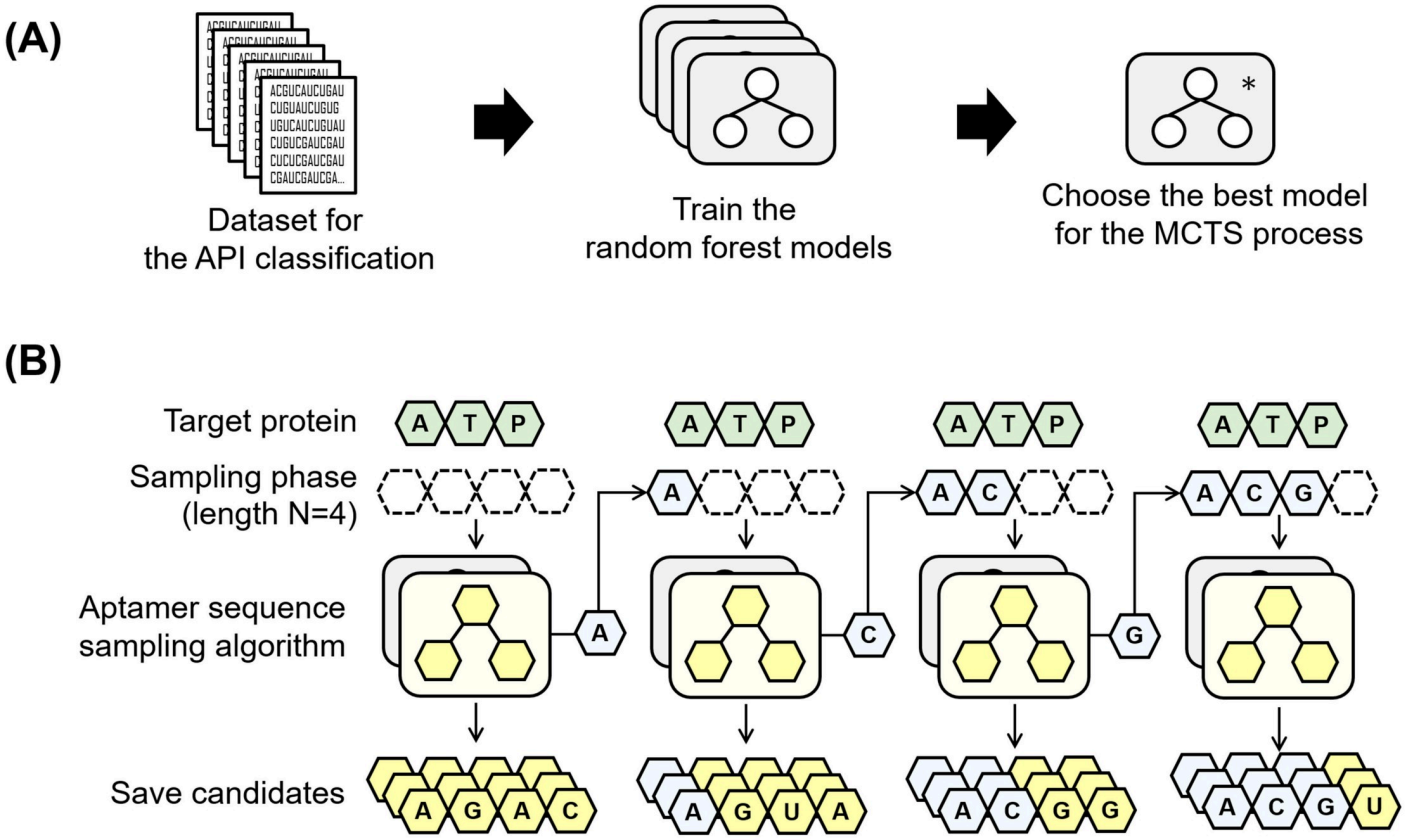
Overview of our study for sampling candidate aptamer sequences using the API classifiers and MCTS. (A) shows the process of choosing the best model from the random forest classifier trained by the API classification benchmark dataset. (B) illustrates our iterative forward sampling algorithm to obtain the candidate aptamer sequences that bind to the given target protein. The sampling algorithm repeats *N* times, where *N* is a user-specified aptamer sequence length. The algorithm takes inputs as the previously selected bases, specifically a target protein sequence and score function that is the best model from (A) before iteration.

We applied a random forest [31] model that has been successfully used for classification and prediction problems when a small volume of training data is available [32]. We trained the model with feature vectors using the scikit-learn package [33]. Because our datasets are imbalanced in terms of the ratio of positive and negative examples, we used “class_weight” parameter of random forest classifier in the scikit-learn package to resolve this data imbalance issue by automatically adjusting weights. As Fig 1A depicts, our models are constructed as a set of multiple random forest models that consist of decision trees for a given API dataset.

### Iterative forward sequence search algorithm based on the Monte-Carlo tree search for generating aptamer sequences

The API classifier takes the encoded feature vectors for a target protein sequence and an aptamer as input. In our original problem, the aptamer sequence (*i.e*., the aptamer) was unknown. We approached this problem by searching for an aptamer sequence that maximizes the output of the API classifier for a given target protein sequence. The unknown part (*i.e*., the aptamer sequence) in the encoded feature vectors for the API classifier can be determined by random sequence sampling. This requires exponential time complexity for searching all combinations of aptamer sequences.

**Fig 2:**
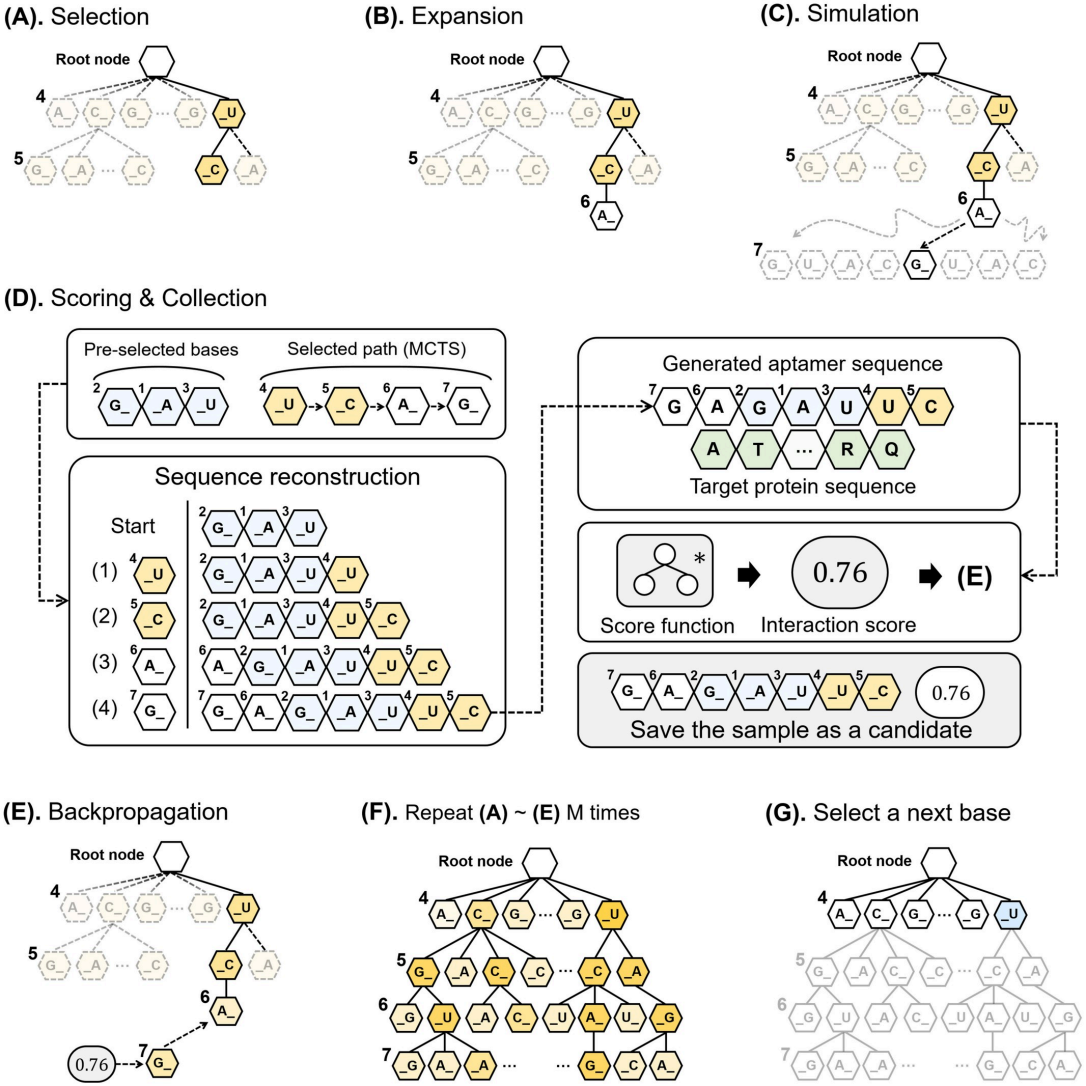
Details underlying our iterative forward sampling algorithm using MCTS. The process (third iteration in terms of total *N*) illustrates the internal process of our sampling algorithm. (A) Selection stage of the MCTS: our method uses previously selected bases in the current sampling iteration. It searches based on the UCT score recursively and finishes after arriving at an unknown position. (B) Expansion stage: a new child node is added in the arrived node randomly from the eight child nodes. (C) Simulation stage: the algorithm attempts a random walk until tree depth *N* and pursues the path from root node to the leaf node. (D) The previous bases (1, 2, 3) and bases of the path (4, 5, 6, 7) are reconstructed as a candidate aptamer sequence. The sequences and interaction scores are added into a set of candidate aptamers. (E) MCTS updates the parameters of the tree using the score calculated in (D). (F) MCTS algorithm repeats the process of (A) to (E) *M* times. (G) The optimal base is selected from the child nodes of a root node for the next sampling iteration.

To reduce the time necessary for search time, we designed our own sequence generation model that recommends candidate sequences without full search. We let the API classifier be denoted as *f*(⋅), the sample sequence length as *N*, and the target protein sequence as *P*. The classifier, *f*(⋅), helps reduce the entropy (uncertainty). The entire process requires *N* iterations, as described in Fig 1B. In the i^th^ iteration, our model generates a set of candidate aptamer sequences with their own binding affinity scores that are calculated by the API classifier. In addition, our model narrows down the search space by adding one base into the blanks of the candidate sequences in each iteration, that is, the bases that have been added to the aptamer candidates are fixed and the rest of the sequences are predicted using MCTS. The prediction steps based on the MCTS are illustrated in Fig 2. The MCTS generates the undetermined bases of the candidate sequences using a path search through a given tree structure that represents the whole sequence search space, as illustrated in Fig 2.

The depth of the tree is *N* when the length of the RNA aptamer sequence is *N*. A path from the root to a leaf determines an aptamer sequence. Each node contains an aptamer base that consists of eight types: A_, C_, G_, U_, _A, _C, _G, and _U. ‘_’ specifies a position where the next sequence base of the child node is placed on the basis of the sequence bases determined by a path from the root to current node. Suppose *N* = 7 and the previously determined bases are “GAU”. If the sampled bases via the MCTS are [_U, _C, A_, G_], the output sequence of this case is generated as follows: GAU → GAUU → GAUUC → AGAUUC → GAGAUUC. If we do not use ‘_’, the bases added to child nodes, which are generated via the MCTS, are placed only either ahead of the previously determined bases or after them. To permit more diverse candidate sequences, we used ‘_’.

Our sampling algorithm that produces the undermined bases in the tree structure consists of five steps during the MCTS: selection, expansion, simulation, scoring and collection, and backpropagation, as portrayed in Fig 2. In the selection step (Fig 2A), a searching path from the root to a leaf is selected based on UCT (Upper Confidence bounds applied to Trees) scores [34], which are formulated as follows:

$$\begin{array}{c} UTC_{i,s_{i},n_{i},N_{i}}\overset{\text{def}}{=}\frac{s_{i}}{n_{i}}+C\times \sqrt{\frac{ln(N_{i})}{n_{i}}} \end{array}$$ (1)

where *i* is a node identifier; *s*_*i*_ is a cumulated exploitation score for the *i*-th node, which is calculated in the backpropagation step; *n*_*i*_ is the number of visits for the *i*-th node; *N*_*i*_ is the number of visits for the parent node of the *i*-th node; and *C* is an exploration parameter.

We set parameter *C* as $\frac{1}{\sqrt{2}}$, as suggested by Chaslot *et al*. [24]. In the expansion step (Fig 2B), a random new child node is added to the end node of the selected path. In the simulation step (Fig 2C), a random playout is performed from the new child node until the path reaches a depth limit. An aptamer sequence candidate that corresponds to the path determined in the simulation step is reconstructed, as illustrated in Fig 2D. This aptamer sequence and the target protein are fed into the API classifier model, and their interaction score is computed using the API classifier. In the backpropagation step (Fig 2E), the parameters of the UCT score at each tree node are updated using the interaction score. These five steps are repeated *M* times, as illustrated in Fig 2F (note that *M* is set to 5000 by default, and this can be adjusted by users). After this iteration, a node that shows the highest score in the child nodes of the root is added to the pre-selected bases, that is, a blank in unknown bases of the aptamer sequences is eliminated (Fig 2G). These updated bases become pre-selected bases for the next round.

After finishing all *N* rounds, our model generates *N* ⋅ *M* candidate RNA aptamer sequences with their own scores. To reduce redundant candidates that exhibit identical RNA secondary structures, we predicted the secondary structures of all our candidate aptamer sequences using ViennaRNA Package 2.0 [35] (note that we determined that two structures were identical when their secondary structures were represented in exactly the same way in terms of the dot-bracket notation). When certain candidates had the same structure, we chose one with the highest score among them. After this post-processing step, we obtained a final list of aptamer candidate sequences as a result of our aptamer generation model. These candidates are sorted by the interaction scores.

### Selecting an API score function among multiple classifiers

Matthew’s correlation coefficient (MCC) is widely used in biomedical applications when datasets are imbalanced [36]. We trained classification models multiple times (2,000 by default) and chose one with the best performance based on MCC for an API score function (note that this classifier selection includes the choice of an optimal encoding method for input sequences). In this procedure, the number of decision trees in the random forest algorithm was set as a random number between 30 and 200. When multiple models were tied in terms of MCC, we selected one that had the fewest number of decision trees. As the score function is repeatedly used with our method, a slim model that has the fewest decision trees reduces computation time during the MCTS.

### Performance evaluation of our classification model

The API classifiers were trained using the random forest algorithm with the binary classification dataset of Li *et al*. [18] and Lee and Han [21]. We validated our trained model by measuring the prediction sensitivity (Sen), specificity (Spe), accuracy (Acc), Youden’s index (J), and MCC as follows:

$$\begin{array}{c} Sn=TP/(TP+FN) \end{array}$$ (2)

$$\begin{array}{c} Sp=TN/(FP+TN) \end{array}$$ (3)

$$\begin{array}{c} Acc=(TP+TN)/(TN+FP+FN+TP) \end{array}$$ (4)

$$\begin{array}{c} J=Sn+Sp-1 \end{array}$$ (5)

$$\begin{array}{c} MCC=\frac{\left(TP*TN-FP*FN\right)}{\sqrt{\left(TP+FP)(TP+FN)(TN+FP)(TN+FN\right)}} \end{array}$$ (6)

where TP, TN, FP, and FN represent true positives (number of pairs predicted as true for real aptamer-protein pairs), false positives (number of pairs predicted as true for wrong aptamer-protein pairs), true negatives (number of pairs predicted as false for wrong aptamer-protein pairs), and false negatives (number of pairs predicted as false for true aptamer-protein pairs), respectively.

### Validation of our generative model using a docking simulation

We obtained top-k candidate aptamer sequences that bind a given target protein using our generative model. To validate the molecular binding affinity of the candidate sequences and protein, we used ZDOCK, which is a computational simulation tool for measuring molecular interactions (note that the docking simulation was not used in the generation of candidate sequences in Apta-MCTS). ZDOCK is commonly used in many molecular interaction studies, such as the theoretical molecular interactions between aptamers and HMG-box Pf [37].

To implement the docking simulation, the three-dimensional structural information of both the input aptamer sequence and target protein is required. Specifically, we converted the sequences into three-dimensional structures. The three-dimensional structures of the RNA aptamers were predicted using SimRNA [38] and the RNAComposer webserver [39]. For some target proteins, their structures were collected from the PDB. When the structure of a certain target protein was unknown, its three-dimensional structures were rendered using the Swiss-Model pipeline, which is a homology modeling method [40]. When several structures were suggested for the target protein, we selected the best structure based on the QMEAN score (> −4.0) [41]. When QMEAN scores were tied, the sequence identity (> 80%) was used as a tie-breaker [40]. More information of target proteins used for this validation are available in S1 Table.

## Results and discussion

### Constructing our aptamer generation models using two training datasets

We constructed our model using two different datasets (listed in Table 1). Our model Apta-MCTS has a score function for the MCTS algorithm, and the score function is replaced by the API classifier. Owing to their varying negative samplings, we had two different API classifiers. Consequently, our model, Apta-MCTS, generates candidate aptamers for a given target protein using the two score functions (API classifiers). For instance, when generating the top five candidate aptamers from Apta-MCTS, 10 candidates are predicted (top five from score functions), and when obtaining candidate sequences according to the sampling method used by Lee and Han [21], the two score functions were applied identically.

### Evaluation of RNA aptamer sequences predicted by our model using six known aptamer-protein pairs using ZDOCK docking simulation

We designed a generative model, Apta-MCTS, that predicts candidate RNA aptamer sequences for a given target protein using the API classifier and MCTS. We generated the top five aptamer candidates using Apta-MCTS with each trained model, that is, 10 aptamer candidates in total using two trained models for a given target protein. To examine the quality of the candidate sequences, we applied the ZDOCK docking simulation [42] and visualized the binding sites of our candidate aptamers for the target protein molecular structure.

**Fig 3:**
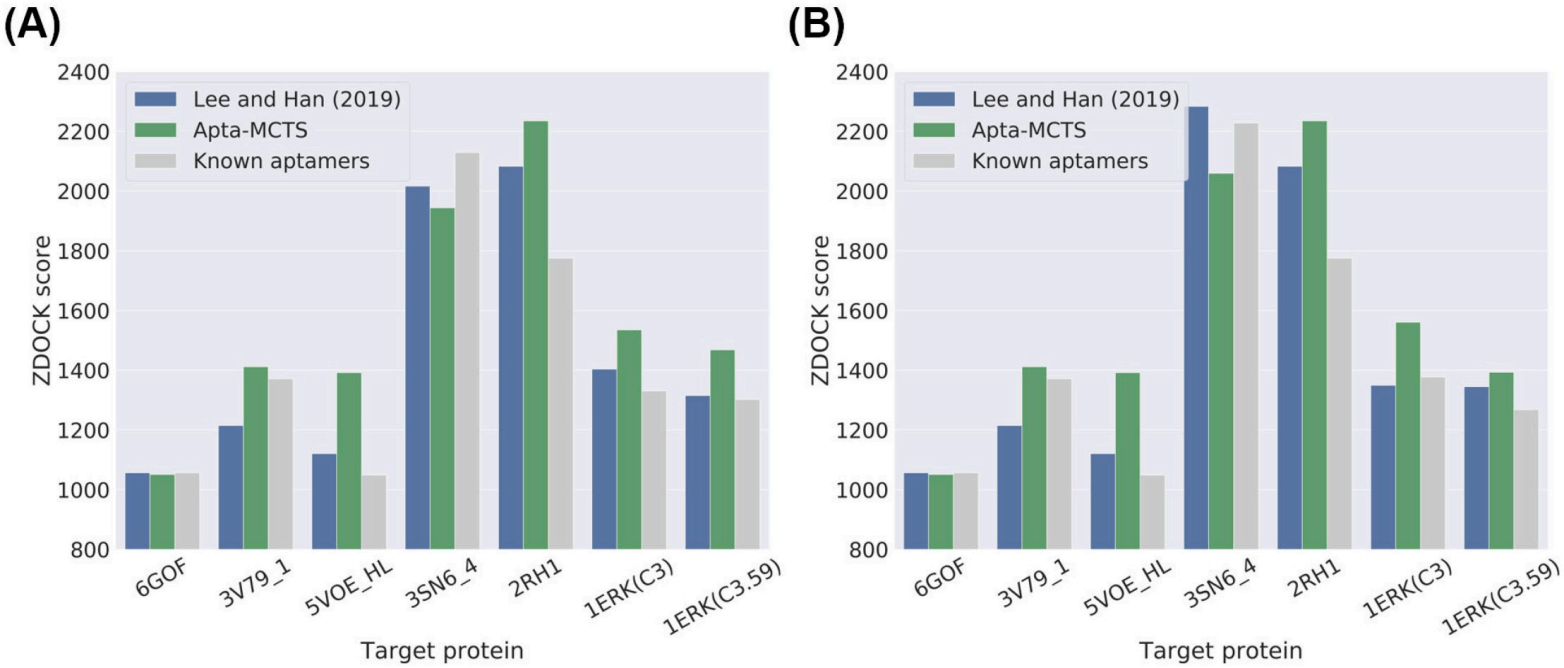
Evaluation of aptamer sequence generation in terms of binding affinity with six target proteins using ZDOCK docking simulation. (A) ZDOCK scores of aptamers using protein structures from the PDB and (B) ZDOCK scores using structures built via the Swiss-Model server. For protein 1ERK, there are two known aptamers, C3 and C3.59, with 90 and 59 bases, respectively. We compared the docking scores of aptamer sequences generated by our model (in green bar) and Lee *et al*. [21] (in blue); the known aptamers (in gray) are listed in Table 2. The candidates of our Apta-MCTS (in green bar) yielded higher docking scores than the results reported by Lee *et al*. [21] (in blue) and the known aptamers (in gray) for five cases: 3V79_1, 5VOE (chain H and L), 2RH1, 1ERK(C3) and 1ERK(C3.59). Additional details are available in S2 Table for (A) and S3 Table for (B).

For this validation, we downloaded six target proteins, with known aptamers, from the PDB. We also obtained the structures of these six target proteins using the Swiss-Model server. The aptamer sequence for 5VOE (chain H and L) is available in PDB [43]. For the rest, their aptamer sequences were obtained via SELEX experiments (conventional SELEX) or previous studies [44, 45]. The list of these six proteins and their aptamers is presented in Table 2 and S1 Appendix. For these six target proteins, we predicted their candidate aptamer sequences using our Apta-MCTS pipeline. We compared our predicted aptamer sequences with the known aptamers in terms of their docking scores, as shown in Fig 3. For this comparison, we chose an aptamer that yielded the highest docking score with the given target protein structure. We set the length of the aptamer sequence in our prediction model to be equal to that of the known aptamers such that we could easily examine their binding affinity through the docking simulation. In addition to the known aptamers, we also compared our candidate sequences with those obtained by Lee and Han [21] (notably, we applied our API classification score to sort the candidate aptamers generated by Lee and Han [21]). For protein 1ERK, there are two known aptamers, C3 and C3.59, with 90 and 59 bases, respectively. We generated two different sets of aptamers using our Apta-MCTS model with 90 and 59 bases, respectively. As a result, our candidate aptamers showed higher docking scores with their target proteins compared to the candidates generated by Lee and Han [21] and the knwon aptamers for five cases (3V79_1, 5VOE_HL, 2RH1, 1ERK(C3) and 1ERK(C3.59)), as illustrated in Fig 3. Fig 3A shows our validation results using protein structures from the PDB and Fig 3B from the Swiss-Model server. There is no significant difference between these two kinds of structures. This shows that our model can generate aptamers that are potentially more suitable for target proteins than the known aptamers.

**Table 2:** Target proteins and aptamers obtained from PDB database, which were applied for our model.

Protein name	Protein PDB-ID	Aptamer ID	Proteins	Aptamers
GTPase KRas	6GOF	V1,V2,V9,D1	[48]	Our own experiments
Neurogenic locus notch homolog protein 1	3V79_1	CS1,…,CS7	[49]	Our own experiments
Coagulation factor X	5VOE(chain H and L)	5VOE:A	[43]	[43]
Endolysin, Beta-2 adrenergic receptor	3SN6_4	A1,A2,A13	[50]	[44]
Beta-2-adrenergic receptor/T4-lysozyme chimera	2RH1_1	A16	[51]	[44]
Extracellular regulated kinase 2	1ERK_1	C3,C3.59	[52]	[45]

Note that the information of aptamer-IDs is available in S1 Appendix.

In addition, we examined whether there is any difference of our generation model working with globular or membrane proteins. Our generative model showed the highest ZDOCK scores in both protein types on average (S1 Fig). Interestingly, ZDOCK docking scores tend to be higher with membrane proteins than with golbular proteins.

**Fig 4:**
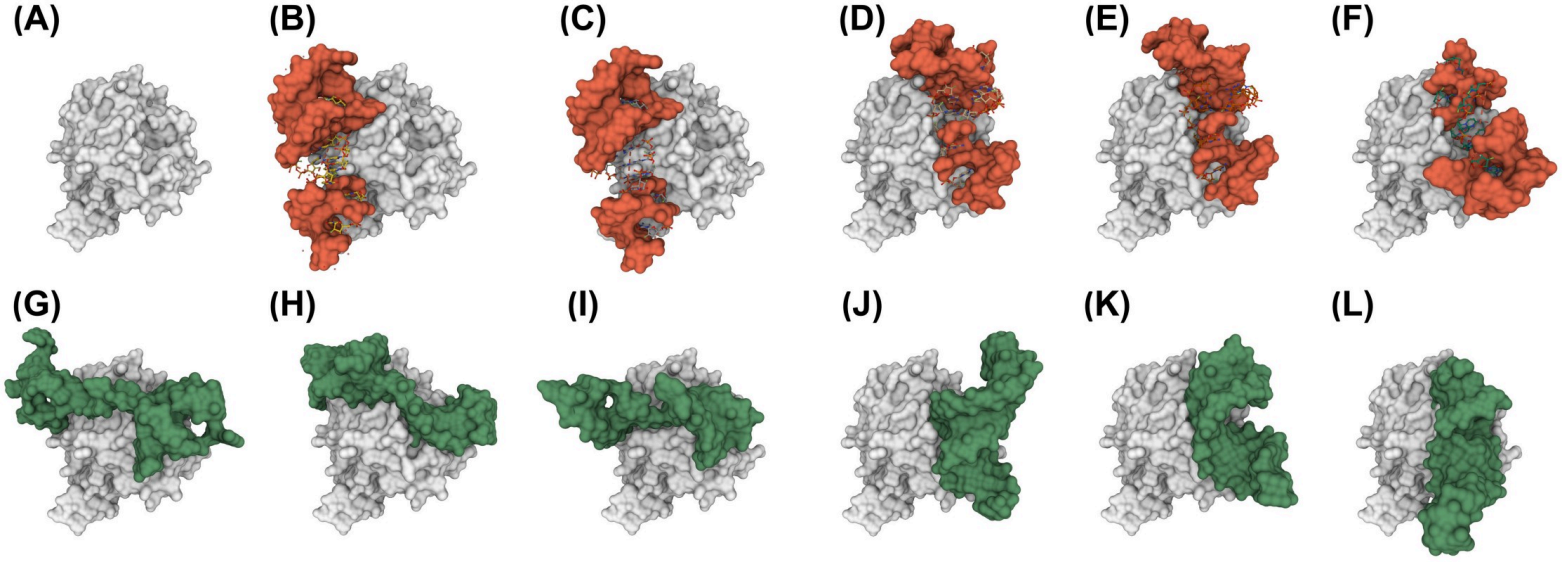
Comparison of binding positions between known aptamer 5VOE(chain A) depicted as a red structure in (B-F) and our candidate aptamers (green) in (G-L) for target protein 5VOE (gray) in (A-L). The structures of aptamers were predicted by SimRNA and RNAComposer and rendered using NGL viewer after the ZDOCK docking simulation was applied. (A) Target protein 5VOE and the angle is always fixed in other figures. (B) Crystallized pose of aptamer 5VOE:A. (C-F) Docked poses of aptamer 5VOE:A with RMSD 5.27Å, 34.31Å, 34.5Å, and 42.33Årespectively compared to the crytallized pose in (B). Our candidates (G-L) show similar binding positions compared to the upper binding sites of (B-F). Especially binding positions in (J-L) are quite similar to ones in (D-F).

For protein 5VOE (chain H and L), the structure of the known aptamer (5VOE:A) is available from PDB. We compared the structures of our candidate aptamers with the known structure of 5VOE:A in terms of their docking positions, as illustrated in Fig 4. The complexes of the docking simulation were generated using ZDOCK and rendered by NGL viewer [46], as shown in Fig 4. Fig 4A shows the structure of the target protein, 5VOE (chain H and L). Fig 4B displays the crystallized pose of aptamer 5VOE:A and Fig 4C–4F four docked poses of 5VOE:A, which were obtained by ZDOCK simulation. As shown in Fig 4C–4F, most aptamers tend to bind to different positions of Fig 4A comparing to the crystallized pose (Fig 4B) except the docked pose in Fig 4C. Fig 4G–4L depict the structures of our candidate aptamers. Interestingly, the binding positions of three candidate aptamers in Fig 4G–4I matched the upper parts of the crystrallized and docked poses. The rest candidates in Fig 4J–4L showed quite similar positions to the docked poses in Fig 4D–4F. In General, the docking process involves two steps: (1) prediction of conformation, position, and orientation of the ligand and (2) assessment of the binding affinity [47]. Therefore, the highly scored candidate aptamers placed at similar sites with respect to the known aptamers indicate that our prediction model has strong potential for suggesting candidate aptamers that interact with the given target proteins.

### Evaluation using benchmark datasets of API pairs

**Fig 5:**
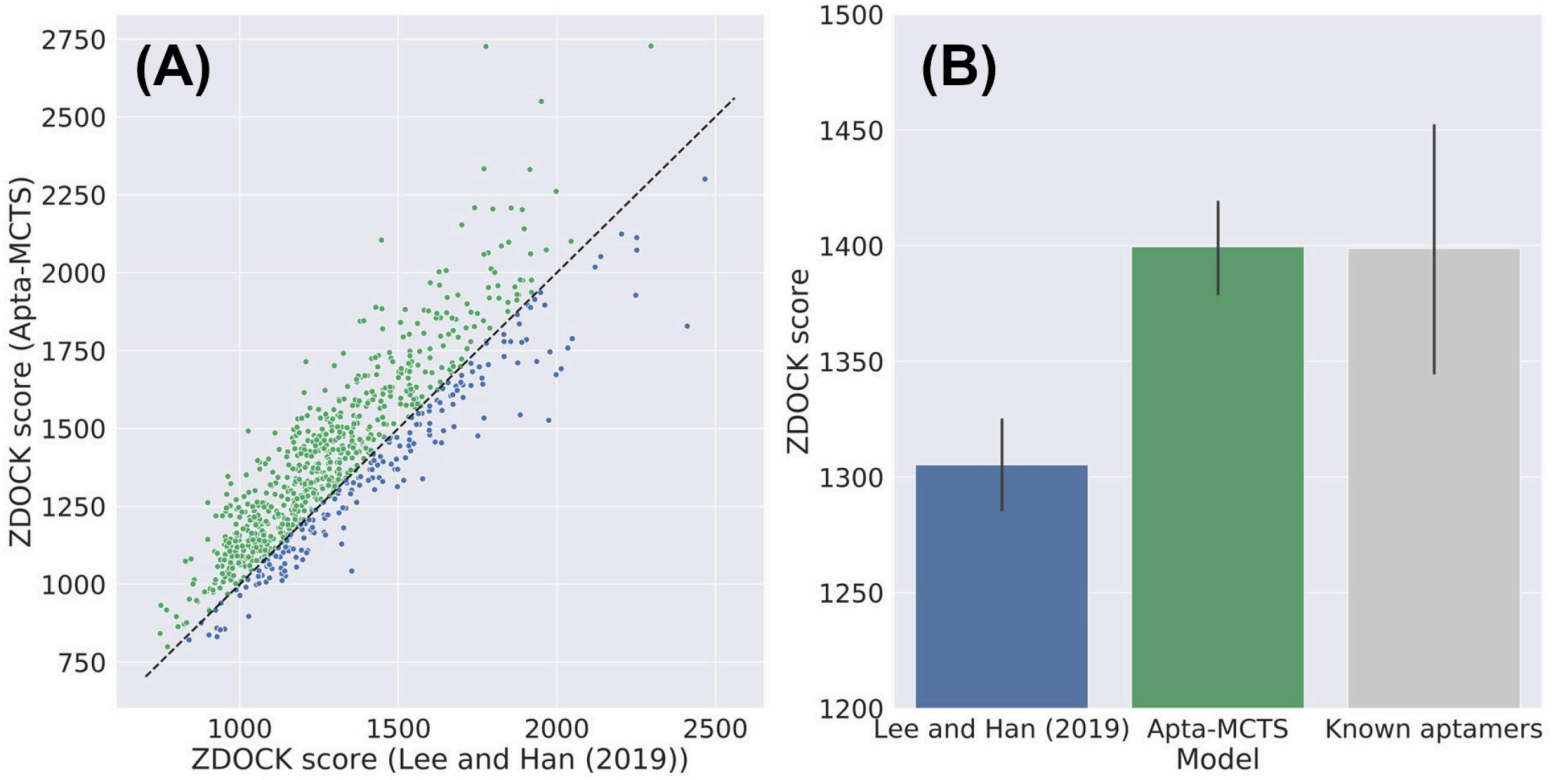
Binding affinity of aptamer samples that have same length with the known aptamers using the docking simulation score by ZDOCK. (A) Comparison of docking scores between our Apta-MCTS and the method employed by Lee and Han [21]. The diagonal dashed line indicates that the docking scores of both models are tied. Green dots above the diagonal line refer to how Apta-MCTS generated better aptamers with higher docking scores than the method used by Lee and Han [21]. (B) Comparison with known aptamers. Apta-MCTS showed the highest docking scores for this comparison.

We evaluated our Apta-MCTS model using 56 positive RNA aptamer-protein sequence pairs in the test dataset of Lee and Han [21]. We generated candidate aptamers that had the same length as the 56 aptamers in the dataset. For each case, we chose the top 10 aptamer candidates according to API classification scores and compared them with the outcomes reported by Lee and Han [21] and known aptamers in the test dataset in terms of docking scores for each pair of a target protein structure and an aptamer candidate sequence. Fig 5A illustrates the comparison of ZDOCK scores between our model and that of Lee and Han [21]. Compared to the model of Lee and Han [21], our model showed better ZDOCK scores for 77% of the total aptamer-protein pairs, as shown in Fig 5A (notably, 77% of the total points in the scatter plot appear above the diagonal line). We summarized all these docking scores as a bar plot and compared them with the known aptamers in the test dataset in Fig 5B. Interestingly, our results showed slightly higher docking scores than the known aptamer-protein pairs. All the scores according to the ranks are available in S4 Table.

### Generating aptamer sequences of various lengths for target proteins

**Fig 6:**
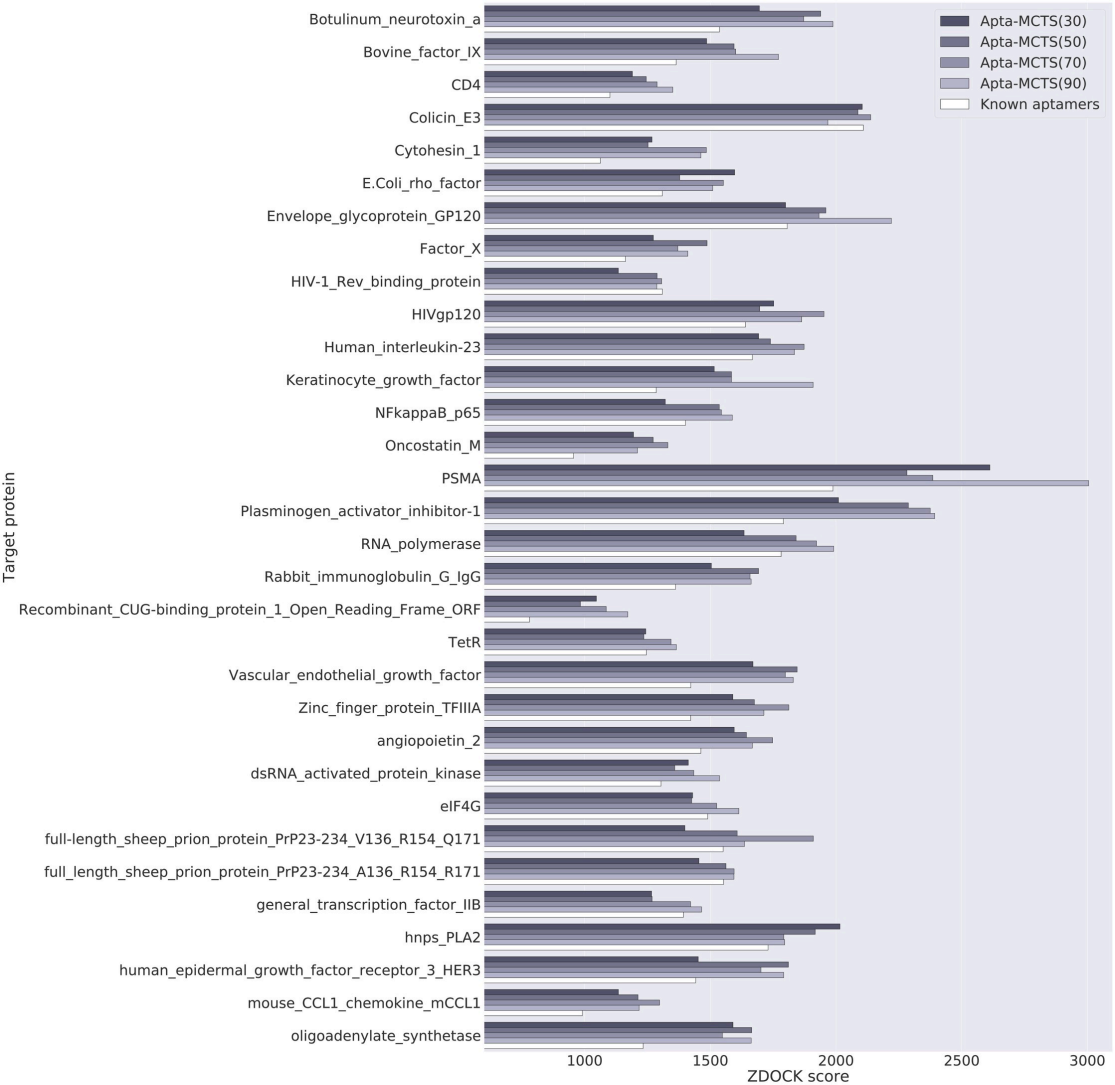
Docking scores for aptamers of various lengths with 32 target proteins in the test dataset. In general, our model generated better aptamers than the known aptamers in the test dataset. The docking scores of our candidate aptamers are reflected by the grey bars, while the known aptamers by the white bars. For most proteins, aptamers with 70 bases and 90 bases (the 3^rd^ and 4^th^ bars in each protein) showed the highest docking score (Note that all the results in detail are available in S5 Table).

Unlike the Lee and Han model [21], our Apta-MCTS generative model enables the generation of candidate aptamers of various lengths. For 32 proteins in the benchmark test dataset, we generated candidate aptamers of various lengths (30, 50, 70, and 90, respectively). Fig 6 illustrates how the docking scores change according to aptamer length. For 14 proteins, aptamers with 90 bases had the highest docking scores. Interestingly, aptamers with 70 bases for 10 proteins had higher docking scores than those with 90 bases. For the five proteins, aptamers with 50 bases had the highest docking scores. In general, the docking scores of aptamers predicted by our model were higher than those of the known aptamers in the test dataset. Notably, the average length of the known aptamers was 51 bases, with a standard deviation of 24.79. This demonstrates that our model could search for an aptamer length that provides high binding affinity with a given target protein.

### Performance evaluation of various encoding methods for input sequences

We used classification models for a score function required in our generative model. For the classification models, input aptamer and protein sequences were encoded. There are 54 combinations of encoding methods for aptamer and protein sequences. To select an optimal encoding method for input sequences, we trained 2000 random forest classifiers for each encoding combination and compared the performance of all the encoding combinations. We summarized this performance comparison in Table 3 for the dataset of Li *et al*. and Table 4 for the dataset of Lee and Han [21]. We abbreviated some of the overall results to Tables 3 and 4 for four protein encoders [CTD, DPC, iCTF, and TPC] and four aptamer encoders [TAC, iCTF, and PseKNC with k = 2, 3] which performed well on both datasets (note that the all experiment results are available in S6 and S7 Tables). According to MCC, a combination of TPC for protein sequences and PseKNC (k = 3) for aptamer sequences showed the best performance for the dataset of Li *et al*., and TPC and PseKNC (k = 2) for the dataset of Lee and Han [21].

**Table 3:** Performance evaluation of various input encoding methods using the dataset of Li *et al*. [18].

API classifier	Protein encoder	Aptamer encoder	Sensitivity	Specificity	Accuracy	Youden’s Index	MCC
Ranfom Forest	CTD	iCTF	0.245	0.999	0.811	0.244	0.439
	CTD	PseKNC(k = 2)	0.304	0.988	0.817	0.292	0.453
	CTD	PseKNC(k = 3)	0.258	0.999	0.814	0.257	0.451
	CTD	TAC	0.174	0.987	0.784	0.161	0.31
	DPC	iCTF	0.259	0.999	0.814	0.258	0.453
	DPC	PseKNC(k = 2)	0.319	0.987	**0.82**	0.305	0.463
	DPC	PseKNC(k = 3)	0.272	0.999	0.818	0.272	0.465
	DPC	TAC	0.195	0.984	0.787	0.179	0.325
	iCTF	iCTF	0.259	**1**	0.814	0.259	0.454
	iCTF	PseKNC(k = 2)	0.319	0.987	**0.82**	0.306	0.463
	iCTF	PseKNC(k = 3)	0.271	0.999	0.817	0.271	0.464
	iCTF	TAC	0.196	0.984	0.787	0.18	0.327
	TPC	iCTF	0.257	0.998	0.813	0.255	0.447
	TPC	PseKNC(k = 2)	0.334	0.975	0.815	0.309	0.441
	**TPC**	**PseKNC(k = 3)**	0.287	0.996	0.819	0.283	**0.467**
	TPC	TAC	0.216	0.972	0.783	0.187	0.308
[18]			0.483	0.871	0.774	**0.354**	0.372
[19]			**0.738**	0.713	0.719	0.451	0.398

The bold font denote the best result in each performance metric. According to MCC (which is commonly used when datasets are imbalanced), TPC+PseKNC(k = 3) was selected as our final choice of encoders. All the results in detail are available in S6 Table.

**Table 4:** Performance evaluation of various input encoding methods using the dataset of Lee and Han [21].

API classifier	Protein encoder	Aptamer encoder	Sensitivity	Specificity	Accuracy	Youden’s Index	MCC
Ranfom Forest	CTD	iCTF	0.862	0.516	0.689	0.379	0.405
	CTD	PseKNC(k = 2)	0.842	0.537	0.69	0.379	0.399
	CTD	PseKNC(k = 3)	0.855	0.52	0.687	0.375	0.399
	CTD	TAC	0.715	0.6	0.658	0.315	0.319
	DPC	iCTF	0.933	0.454	0.693	0.387	0.442
	DPC	PseKNC(k = 2)	0.928	0.474	0.701	0.402	0.452
	DPC	PseKNC(k = 3)	0.932	0.462	0.697	0.394	0.448
	DPC	TAC	0.881	0.561	0.721	0.442	0.469
	iCTF	iCTF	0.931	0.493	0.712	0.424	0.473
	iCTF	PseKNC(k = 2)	0.949	0.499	0.724	0.448	0.502
	iCTF	PseKNC(k = 3)	0.952	0.498	0.725	0.45	0.506
	iCTF	TAC	0.887	0.567	0.727	0.454	0.481
	TPC	iCTF	0.931	0.459	0.695	0.389	0.443
	**TPC**	**PseKNC(k = 2)**	**0.997**	0.466	0.731	0.463	**0.546**
	TPC	PseKNC(k = 3)	0.986	0.466	0.726	0.452	0.53
	TPC	TAC	0.978	0.492	**0.735**	**0.47**	0.538
[21]			0.768	**0.661**	0.714	0.429	0.431

The bold font denote the best result in each performance metric. According to MCC, TPC+PseKNC(k = 2) was selected as our final choice of encoders. All the results in detail are available in S7 Table.

Our results revealed that our two classifiers have different characteristics. While our first classifier has high specificity, the second has high sensitivity. Comparing to Li *et al*. [18], Zhang *et al*. [19], and Lee and Han [21], our classifiers with the selected encoding methods were good enough to apply for our generation model. Since we used both classifiers with different characteristics for our generation model, it enables Apta-MCTS to obtain various candidate aptamers from a broad perspective.

## Conclusion

In the present study, we developed a generative model, called Apta-MCTS, to determine potential RNA-aptamer candidates for a target protein of which only the sequence is available. While recent classification studies on nucleotide sequences that bind to target proteins have focused on the performance of binary classification, only a few studies have attempted to determine candidate aptamers. We designed a machine learning approach that generates candidate RNA-aptamers based on a discriminative classifier of API and MCTS. To feed features from input data properly to our model, we applied the TPC and PseKNC encoders. The scores required for MCTS were computed using our API classifiers based on random forest model. To evaluate Apta-MCTS, we simulated the binding affinity of our candidate aptamers and target proteins based on their molecular structures with ZDOCK. In general, Apta-MCTS yielded higher docking scores than known aptamers as well as compared to results from other generation methods. Our model can generate aptamer sequences of any length that users wish to build. We investigated the effect of aptamer lengths for given target proteins. Aptamers of 70–90 bases improved docking scores compared to known aptamers. All these results show that our Apta-MCTS can produce aptamer sequences that are more appropriate for relevant experiments than existing methods. There is still some room to improve our generative model. For example, its performance can be increased via a rigorous study for optimizing API classifiers which are used as a score function of the generative model. We believe that our ongoing efforts in this area can substantially reduce the cost and time required for drug discovery using aptamer design.

## Supporting information

S1 Appendix Additional information of known aptamers for six target proteins. (PDF)

S1 Fig Evaluation of our generative model working with globular or membrane proteins. (A) Evaluation of aptamer sequence generation with six target proteins: 6GOF, 3V79 1, 5VOE HL, 2RH1, 1ERK(C3) and 1ERK(C3.59) which were used in Fig 3, and (B) with 32 target proteins used in Fig 5.
(TIF)

S1 Table The details about the three-dimensional structures of the target proteins used for docking simulation. The table includes additional data for the target proteins such as protein name, description, source, and template Swiss-Model threshold values.
(CSV)

S2 Table Docking scores between target protein structures from PDB and candidate aptamers. (CSV)

S3 Table Docking scores between target protein structures from Swiss-Model and candidate aptamers. (CSV)

S4 Table The docking scores of the candidate aptamers for the 32 target proteins ranked by Apta-MCTS and Lee *et al*., [21]. The table contains the ZDOCK docking scores of the top 10 candidate aptamers for each target protein.
(CSV)

S5 Table Docking scores between the 32 target proteins and candidate aptamers generated by Apta-MCTS with various lengths (30, 50, 70, and 90bp). (CSV)

S6 Table Performance comparison of 54 encoding methods using the dataset of Li *et al*., [18]. (CSV)

S7 Table Performance comparison of 54 encoding methods using the dataset of Lee and Han [21]. (CSV)

S8 Table Summary statistics of RNA secondary structure redundancy removed in the post-processing step of Apta-MCTS. The table includes information expressed in terms of parameters of negative binomial distribution for RNA secondary structure redundancy.
(CSV)
