# Supplementary material for: Predicting aptamer sequences that interact with target proteins using an aptamer-protein interaction classifier and a Monte Carlo tree search approach
Source: PLoS One. 2021 Jun 25;16(6):e0253760. doi: 10.1371/journal.pone.0253760 (PMC8232527; doi:10.1371/journal.pone.0253760)
Supplement: S1 Appendix — (PDF) [file pone.0253760.s001.pdf]

S1 Appendix

ID and sequences of the known aptamers in Table 2

Aptamer-ID	Sequence (T→U)
V1	ATTGGGGAAGGCGAGGTTGGGGACGCGGGGCGCGTGGTGT
V2	TGGGGTGAGTGGGCGAGGGGTGAGGAGATGAAGGGCATGG
V9	CAGGGGAGCGGCTGCACGGGTTCTGCGGGGGGAGGTTGGG
D1	ACATGGGGGGACGTCGGTCAGGGTAGGTTAGGGGGAGAGA
CS1	GGTCTCATAGGGTTCAAAGGCATTTGGTGTGAGAGCAGAG
CS2	TGTGGGAATGTAGGCCGTGTTCCCATGCAGAGGCGATCGG
CS3	TAAGGATTGATGGTGTGGGGGCAGTCTGGTCCCATAGGGT
CS4	CAATAATCTAACTGTCCCATGTTTCGGGTGTGTGGGAGAG
CS5	GAAGAGGAATTGACGGTGTGAGTAGGCGTTCTCATAGGGT
CS6	AACCTACAATGGTGTGGGTACCTCCTTCCCATAGGACCCG
CS7	TATGGTCCCAATTGGTGTCCTCTGTAGGGGAGTGGGAGCA
A1	GCAUAACAGCUGGUCCACCGCCGCAUCAAGGCGAGAGCGC
A2	GAUCCACCUGGAGGGGGUACAUCGCGCGGGUCGCCGAUUA
A13	GUCUUAGCUCUGCAGCCCACGGAGGAGAGGGGAGGGCCGA
A16	AACAAAGCAAGGAGGAGUGCUGCCGCAUUCUCGAAGGCCU
C3	GGGAGAGGAGGGAGAUAGAUAUCAACUGCAGGGUAGCACACGAAGGGCACCGUAGCAAUACGGU UGUUCUGGUUAACUUUCGUGGAUGCC
C3.59	GGUAGCACACGAAGGGCAUCGUAGCAAUACGGUUGUUCUGGUUAACUUUCGUGGAUGCC
5VOE:A	GAGAGCCCCAGCGAGAUAAUACUUGGCCCCGCUCUU
