# Supplementary figures and images for: Predicting aptamer sequences that interact with target proteins using an aptamer-protein interaction classifier and a Monte Carlo tree search approach
Source: PLoS One. 2021 Jun 25;16(6):e0253760. doi: 10.1371/journal.pone.0253760 (PMC8232527; doi:10.1371/journal.pone.0253760)

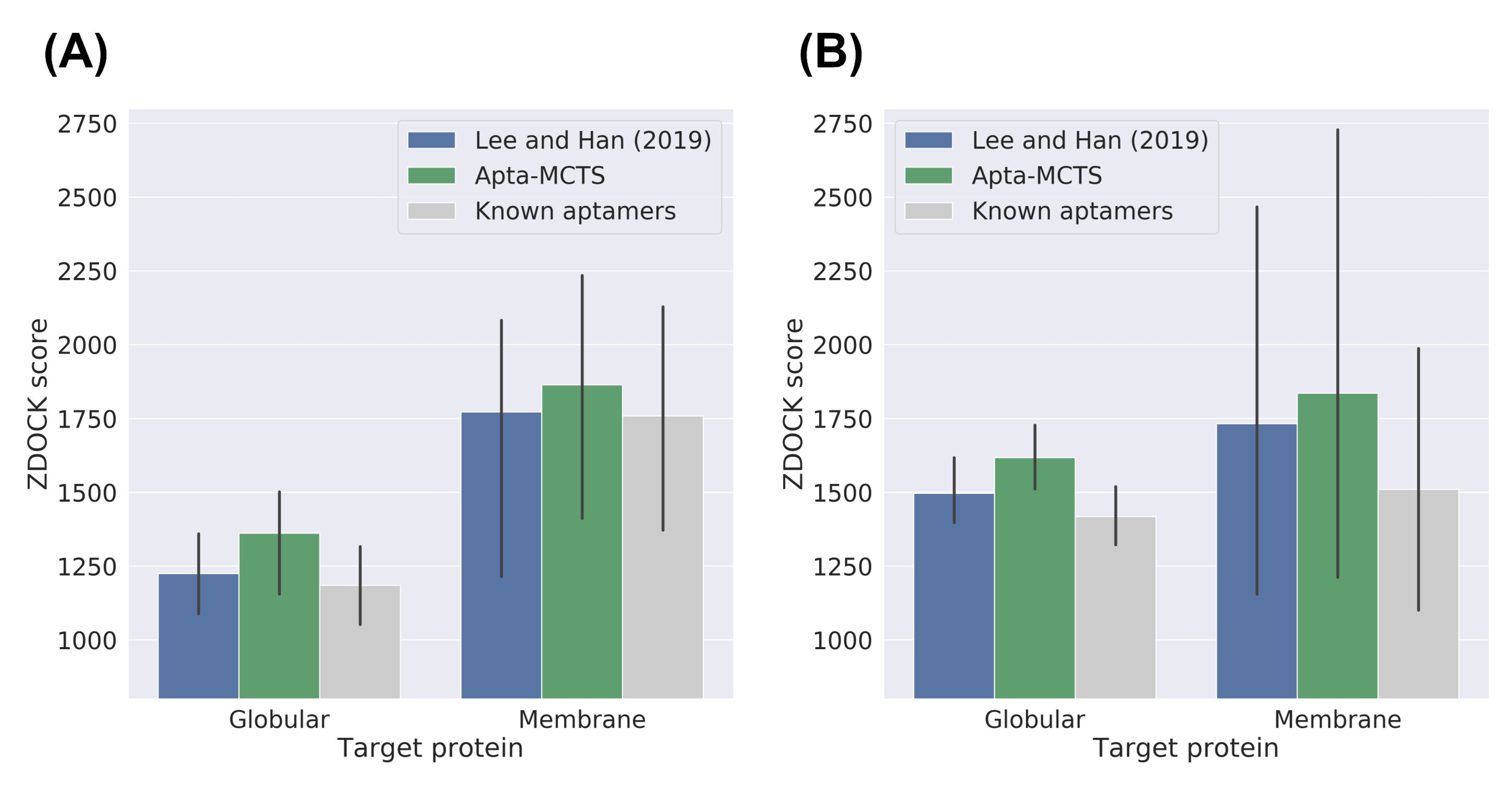

Supplement: S1 Fig — (A) Evaluation of aptamer sequence generation with six target proteins: 6GOF, 3V79 1, 5VOE HL, 2RH1, 1ERK(C3) and 1ERK(C3.59) which were used in Fig 3, and (B) with 32 target proteins used in Fig 5. (TIF) [file pone.0253760.s002.tif]
